# Empirical assessment of seismic design hazard’s exceedance area

**DOI:** 10.1038/s41598-021-98388-9

**Published:** 2021-09-22

**Authors:** Iunio Iervolino, Antonio Vitale, Pasquale Cito

**Affiliations:** Dipartimento di Strutture per l’Ingegneria e l’Architettura, Università degli Studi di Napoli Federico II, Naples, Italy

**Keywords:** Seismology, Civil engineering

## Abstract

Design ground motion intensities determine the actions for which structures are checked, in the conventional approach of seismic codes, not to fail the target performances. On the other hand, due to inherent characteristics of probabilistic seismic hazard analysis (PSHA), it is expected that site-specific design intensity based on PSHA is exceeded in the epicentral area of moderate-to-high magnitude earthquakes. In the context of regional seismic loss assessment and of the evolution of seismic codes from the regulator perspective, it is useful to gather insights about the extent of the zone around the earthquake source where code-conforming structures are expected to be systematically exposed to seismic actions larger than those accounted for in design. To assess such areal extent based on empirical evidence is the scope of the study presented in the paper. To this aim, peak ground acceleration ShakeMap data for Italian earthquakes from 2008 to 2020 were compared to the current design intensities in the same areas for which the maps are available. This allowed, first, to develop simple semi-empirical models of the exceedance area versus the magnitude of the earthquakes. Second, it allowed to model the probability that an earthquake of given magnitude causes exceedance of the design intensity via logistic regressions. Coupling the first and second class of models provides an approximation of the expected exceedance (logarithmic) area upon occurrence of an earthquake of given magnitude. Such an area can be of several thousand square kilometers for earthquakes occurring relatively frequently in countries such as Italy.

## Introduction

In Italy, as well as in other countries of advanced earthquake engineering culture, design actions for seismic design are based on probabilistic seismic hazard analysis (PSHA). In particular, pseudo-spectral accelerations with fixed exceedance return periods (T_r_) are used for performance-based design. It has been extensively shown that, especially in the case of PSHA based on seismic source zones, it is expected that accelerations corresponding to common return periods for design (e.g., 50 and 475 years, that—in the Italian code^1^—refer to *damage* and *life safety* limit states for ordinary constructions, respectively) are exceeded in the epicentral area of moderate-to-high magnitude earthquakes, with the precise minimum magnitude likely to cause exceedance depending on the spectral ordinate being considered and the hazard level of the region of interest^2^. This is an inherent characteristic of PSHA and not necessarily an evidence of fallacy of the hazard analysis. As a consequence, it is expected that exceedance is systematically found in past events. In fact, this happens even for earthquakes with magnitude relatively less than the maximum possible considered in PSHA for the region of interest^3–5^.

Given the site-specific nature of PSHA, these exceedances have been studied at sites where recording stations were operating at the time of the event under scrutiny. On the other hand, it might be worthwhile to investigate the extent of the exceedance area around the earthquake source. This might be useful, for example, for the calibration of the so-called parametric earthquake insurance^6^ and regional seismic loss assessment. Moreover, the development and calibration of seismic codes can also benefit of insights about the extent of the area around the earthquake source where exceedance of the prescribed design intensity is anticipated.

This is the aim of the study herein presented, based on the Italian ShakeMap^7^ data, expressed in terms of peak ground acceleration (PGA), released by the *Istituto Nazionale di Geofisica e Vulcanologia* (INGV) for a few thousands earthquakes occurred between 2008 and 2020. For each of these events, the ShakeMap were compared to the seismic hazard corresponding to two exceedance return periods commonly used for structural design, to determine whether exceedance was caused by the event and, if yes, to estimate the extent of the exceedance area. In turn, the exceedance areas obtained were used to calibrate simple log-linear models as a function of the earthquake magnitude. These models provide an approximation of the expected size of the exceedance logarithmic area, given the occurrence of an earthquake of fixed magnitude, in case it causes exceedance. Separately, using the same data, logistic models were applied to calibrate the probability of causing exceedance, given magnitude. The two models combined provide an approximation of the expected exceedance (logarithmic) area around the source, given magnitude.

To illustrate the study and discuss its results, the remainder of the paper is organized such that ShakeMap data are described and the PSHA results used for the comparison are recalled first. Subsequently, the estimated logarithms of the exceedance areas, when larger than zero, are regressed against earthquake magnitude. At the same time, the magnitude of all earthquakes is used to determine the probability of exceedance given magnitude, via a logistic regression approach. A discussion of the obtained results follows, by virtue of an application of the derived semi-empirical models.

## Data

In Italy, ShakeMap is mainly used for emergency management, that is, to assess the potential relevance of the earthquake for civil defense purposes in near-real-time. In fact, the shaking intensity is evaluated automatically after the ensemble of the national seismic monitoring networks has recorded an earthquake of magnitude equal to or larger than three (a few days later, the ShakeMap is revised, and quality-checked waveforms made available). Eventually, ShakeMap is also often used to relate the observed damage to the built environment to the earthquake, providing an estimated measure of ground motion at the construction site. For example, to derive information about the vulnerability of the damaged structural typologies^8^. Furthermore, in Italy, exceedance of some ground motion thresholds from the ShakeMap at a building site is used in the context of restoration/reconstruction policy for the hit area by the national government; see, for example, the Italian law 122 of 2012 of August 1 2012, according to which, the need for safety verification and retrofitting in the area hit by the earthquake is also based on the sustained shaking as inferred by ShakeMap and the performance of the building.

**Figure 1:**
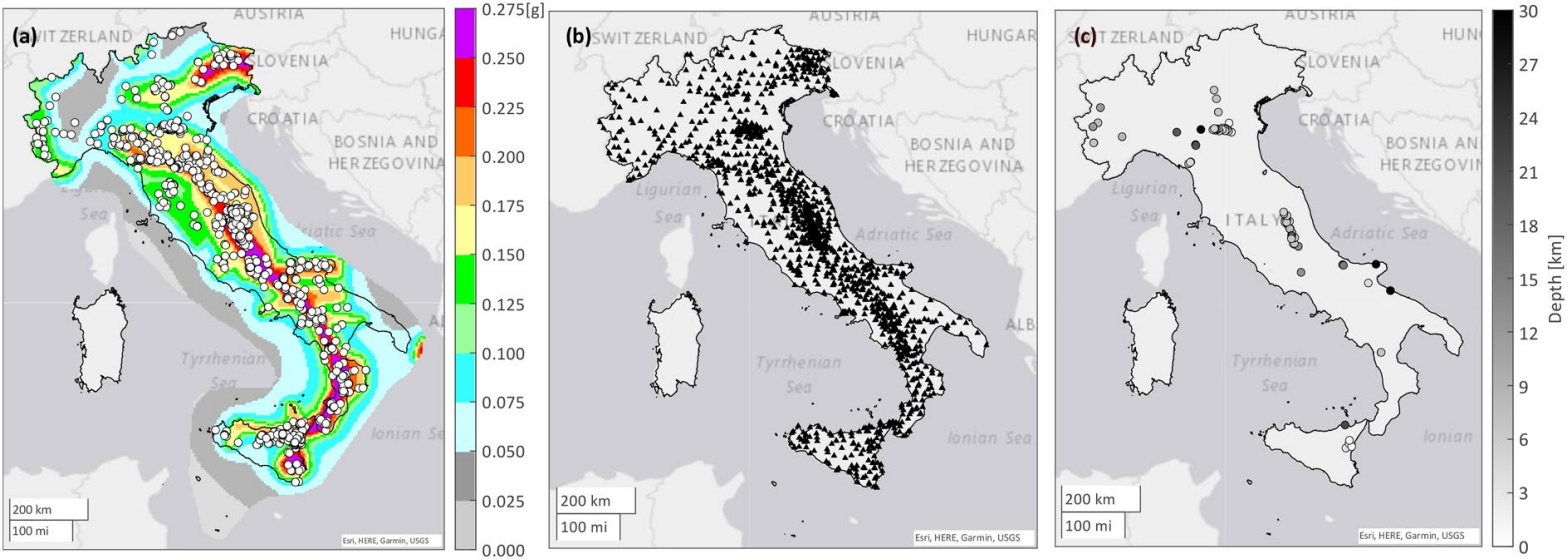
Epicenters of the considered earthquakes on the map of the PGA on rock with T_r_ = 475 years used for seismic design (**a**); recording stations providing the basic data for the ShakeMap computations (**b**); epicenters and depth of the events causing PGA exceedance (**c**). (Maps created via MathWorks MATLAB R2021a; https://it.mathworks.com/products/matlab.html).

The current INGV implementation of ShakeMap in Italy relies on the V4 release of the ShakeMap software^9^. ShakeMap is provided in terms of five ground motion intensity measures, that is, peak ground velocity, PGA, and three 5% damped spectral pseudo-acceleration, and accounts for local soil site conditions according to a large-scale geological model. ShakeMap V4 is based on the multivariate normal distribution approach, according to which the intensity measures, recorded at the sites where instruments are located, are used to estimate unobserved intensities at other sites^10^. Consequently, data include an *error* term for each point at which ground motion intensity is evaluated, which is applicable to the logarithm of the intensity and accounts for the uncertainty associated to the ShakeMap estimation. These data are made publicly available through a dedicated website (see Data availability) and those available from 2008 to 2020 have been considered for the analyses described in the following. In particular, for reasons related to the comparison with the Italian design hazard, only those with the epicenter falling on the Italy mainland are included in the analyses, to guarantee that the ShakeMap falls in a region for which the seismic hazard is available (for this very same reason, the events pertaining to the Sardinia Island are excluded as the PSHA results are not available for this region). In summary, the number of earthquakes initially considered is 3138, with magnitude within the interval [2.9, 6.5] and maximum hypocentral depth equal to 427.4 km. Among these, the events with depth larger than 50 km (49 in number) and those for which the ShakeMap is not based on ground motion recordings within the map area (701 in number) have been excluded, resulting in 2401 earthquakes (13 overlap between the two categories removed). The epicenters of the considered events are represented in Fig. 1a on the map of the PGA on rock with 475 years (yr) exceedance return period (see the next section). As expected, most of the events has occurred where the PGA hazard is the largest. Figure 1b, shows the recording stations within the Italian borders that have provided the recordings to build the ShakeMap of the considered events; they are close to two thousand in number. The average number of recording stations within the ShakeMap area of each event is 54. (Fig. 1c shows those events in Fig. 1a causing exceedance; see the Results section).

**Figure 2:**
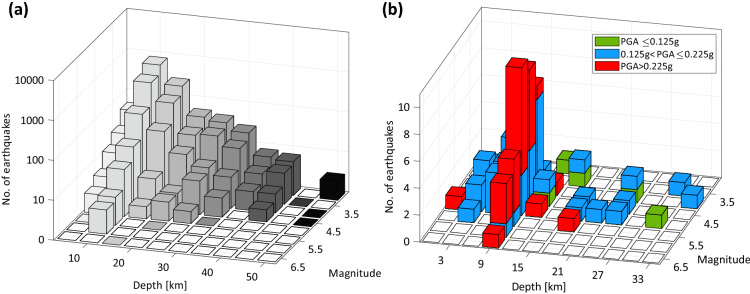
Earthquakes, the ShakeMap of which are considered in this study, described in terms of magnitude and hypocentral depth distribution (**a**); events causing exceedance of the 50 years design hazard in terms of PGA described in terms of magnitude and hypocentral depth, and also in terms of the hazard level of the region were they have occurred (**b**).

To further analyze the input data for the subsequent analyses, Fig. 2a shows the magnitude and depth distribution of the earthquakes. It can be observed that, as expected, the magnitude distribution is pseudo-exponential, that is of the Gutenberg-Richter type^11^. It must also be noted that the three major seismic sequences occurred in Italy in the last decades, which featured a mainshock with magnitude (M) larger than six, are all represented in the dataset; L’Aquila 2009 (M6.3), Emilia 2012 (M6.1), Central Italy 2016 (M6.5). As a consequence, the ShakeMap for fore- and after-shocks of these events are also present among data. They are retained for the calibration of the models developed in this study even if the exceedance refers to classical PSHA, which only accounts for the mainshock events. This is consistent assuming that, given occurrence of the event and its magnitude, both the probability of causing exceedance around the event source and the size of the exceedance area are not systematically different among foreshocks, mainshock, and aftershocks. This choice, considering all available data, maximizes the robustness of the results without introducing any bias for the very same reasons of the customary approach to develop ground motion models^12^ that pools together data from all the mentioned categories of events. In other words, only the occurrence in time and space is systematically different between mainshocks, fore-, and after-shocks, not the effects they produce around their source area, if the magnitude is the same.

## Methods

The Italian building code^1^ prescribes to define seismic actions based on PSHA. Results of the analysis consist of PGA and spectral accelerations, on rock site conditions, for ten vibration periods from 0.1 to 2 s and in range of exceedance return periods from 30 to 2475 years^13^. Herein, the areas in which the exceedance of code-mandated PGA considered for structural design was observed, are delimited with respect to two of these return periods. In particular, 50 years and 475 years are considered, because they are relevant for ordinary constructions in Italy.

PSHA results refer to rock soil site class and data are publicly available (see Data availability). According to the Italian code, to account for local site conditions the PGA on rock must be multiplied by a factor that depends on the local soil site class and (indirectly) the return period of the seismic action considered (in fact, the code also considers modification of design hazard for topographic conditions, such a modification is neglected herein). In this study the PGA on rock from PSHA is modified to account for local site conditions via the same local geological information used to derive the ShakeMap. In fact, the ShakeMap data include, for each site where intensity is estimated in a given earthquake, the soil class of the site according to the same classification scheme of the code, so that seismic action on soil could be consistently evaluated.

Note, finally, that the ground motion models employed to develop the hazard map are not the same as those implemented in ShakeMap, which are more recent. This is expected to have an effect on the results presented in the following, yet not detrimental, because all the models are calibrated on (partly overlapping) Italian ground motion data.

### Earthquake occurrence, exceedance, and hazard level

Before proceeding any further, it must be noted that, given magnitude, the exceedance area depends on the PGA value (i.e., threshold) to be exceeded. To understand this issue, it is useful to define as a lower-hazard region a geographical area where the PGAs corresponding to a fixed return period are systematically lower than those for the same return period in another region, which can be referred to as higher-hazard (see Fig. 1a). Ground motion models tell that, for the same magnitude, the exceedance area is expected to be larger in the former with respect to the latter. To account for this issue, the earthquakes discussed were primarily classified in terms of hazard level at their epicenters. Because the maximum PGA on rock with 475 years return period in Italy, according to the current PSHA model, is below 0.3 g, an earthquake was, arbitrarily, classified as occurring in a low-, mid-, or high-hazard area if the PGA with 475 years return period of its epicenter is equal to or below 0.125 g, between 0.125 and 0.225 g, or above 0.225 g, respectively.

The ShakeMap produced by INGV and used in this study are provided on grids that vary from earthquake to earthquake in terms of location, yet all having a fixed resolution of about 1 km. On the other hand, the Italian building code provides the site-specific data to build the design spectra for a grid covering the whole country and with a resolution of not more than 10 km. Therefore, for the comparison, the design seismic actions at each of the sites of the grid of a given ShakeMap were retrieved via a weighted average of the PSHA data at the four closest points, where the weights are the reciprocal of the distances of the considered site from the four for which the code provides hazard information (in fact, this is the procedure prescribed by the code to define design seismic actions for a site not coinciding with one of the grid points).

## Results

The earthquakes causing exceedance are defined such as those for which, in at least one point of the grid where the ShakeMap is provided, the PGA is larger than the code-defined counterpart (i.e., from PSHA), accounting for the local site conditions as discussed above. The earthquakes for which this condition is met are 78 and 15 when the design actions with 50 years and 475 years return period are considered, respectively (clearly, the latter is a subset of events of the former group). These events, as discussed in the introduction, are mapped in Fig. 1c, where their hypocentral depth is also given. Also, the joint distribution of magnitude and depth of the earthquakes causing exceedance of the PGA hazard with 50 years exceedance return period is shown in Fig. 2b. Most of these events has depth within 10 km from the surface and occurred mostly in what have been defined as mid- and high-hazard areas. This can be appreciated in Table 1, which provides the number of earthquakes causing exceedance as a function of the hazard class.

**Table 1:** Earthquakes causing exceedance area larger than zero, according to ShakeMap, divided per hazard level, based on the PGA on rock with 475 years exceedance return period at the epicenter.

	Tot	PGA ≤ 0.125 g	0.125 g < PGA ≤ 0.225 g	PGA > 0.225 g
T_r_ = 50 years	78	7	44	27
T_r_ = 475 years	15	2	5	8

## Exceedance area given exceedance

The extent of the exceedance area is related to the earthquake magnitude via a simple relationship of the kind:

1 $$E\left[ {\log \left( {Area} \right)\left| {M = m \cap {\text{exceedance}}} \right.} \right] = a + b \cdot m.$$

This model has to be intended as a model fitting the data and no ad-hoc investigation of the functional form was carried out; therefore, no residual term was provided. In any case, if the hypotheses of the linear regression would hold, the equation could be interpreted as providing the expected value of the logarithm of the exceedance area given earthquake magnitude and given that the earthquake causes some exceedance area larger than zero, that is $E\left[ {\log \left( {Area} \right)\left| {M = m \cap {\text{exceedance}}} \right.} \right]$.

**Figure 3:**
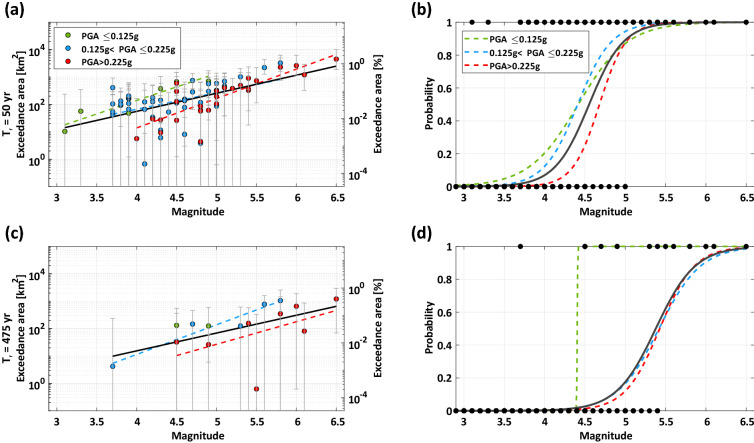
(**a**) PGA exceedance area of the design action with 50 years return period, in terms of km^2^ and of fraction of the Italian territory, as a function of event magnitude; (**b**) logistic regression and data of events exceeding the 50 years return period PGA; (**c**) and (**d**) refer to 475 years return period.

Different fittings were attempted for data divided in hazard classes because, as discussed in the previous section, it is expected that the exceedance area is systematically larger for earthquakes in a lower hazard region, which is actually observed in Fig. 3a,c, where the exceedance data are displayed. Note that two area metrics are considered in the figure: the left axis is the area in km^2^, while the right axis is the area measured as a percentage of the Italian territory. In the figure, a vertical bar is given for each datum, which provides bounds to the estimated exceedance area, obtained adding and subtracting—to the logarithm of the PGA estimated at a point—the associated error term provided by the ShakeMap for that point. However, this uncertainty is not modelled in the regression because, as mentioned, the standard deviation in ShakeMap is point- and earthquake-specific and to properly consider it in the models would require a level of complexity in statistical analysis which is out of the scope of this study.

Table 2 provides the coefficients of Eq. (1) fitted on the data of Fig. 3a,c, where the corresponding lines are also displayed. The coefficients were retrieved separately for the three hazard classes. However, it can also be noted that for some cases the data in a specific hazard class were barely sufficient or insufficient to calculate the coefficients of Eq. (1); therefore, fittings obtained pooling all the data together are also provided as the black solid lines.

**Table 2:** Curve-fitting coefficients for the log of the exceedance area of the design actions as a function of magnitude.

	Tot	PGA ≤ 0.125 g	0.125 g < PGA ≤ 0.225 g	PGA > 0.225 g
**T**_**r**_** = 50 years**				
a	− 2.0599	− 4.1675	− 1.3522	− 7.2615
b	1.5197	2.2758	1.3868	2.4712
**T**_**r**_** = 475 years**				
a	− 3.2520	–	− 7.5351	− 6.2055
b	1.4967	–	2.4918	1.8976

It results that the exceedance area can be as large as some thousands of square kilometers for the largest earthquake in the dataset (i.e., M6.5). Generally, the expected exceedance area increases by an order of magnitude by increasing by a unit the earthquake magnitude. The classification per hazard levels generally shows what anticipated, that is, the exceedance area tends to decrease with the hazard level where the earthquake occurred. However, for some events of similar magnitude the exceedance area is larger for the earthquake where the hazard is also higher. Moreover, for multiple earthquakes of the same magnitude occurring in the same hazard class, differences by one order of magnitude in exceedance area can be observed.

These insights have multiple explanations. First, the fact that the magnitude is, knowingly, a very simple proxy for the effects of an earthquake in a region, so that to the same magnitude very different effects can be observed at the sites because of both source and large-scale propagation effects (this rough explanation motivates, for example, the introduction of an inter-event residual term in ground motion models). Moreover, one must consider that the classification of the earthquakes in hazard classes does not account for the fact that earthquakes can manifest their effect at sites on systematically different soil classes so as to significantly alter the seismic design action thresholds with respect to which evaluate exceedance. Finally, hypocentral depth may also play a role in systematically changing the effects of an earthquake in its epicentral region, all other factors remaining the same.

### Probability of exceedance given magnitude

Because Eq. (1) could possibly be interpreted as the expected value of the logarithm of the exceedance area given magnitude in the case of such an area is larger than zero, the expected value of the log of the exceedance area given magnitude can be obtained as follows:

2 $$E\left[ {\log \left( {Area} \right)\left| {M = m} \right.} \right] = E\left[ {\log \left( {Area} \right)\left| {M = m \cap {\text{exceedance}}} \right.} \right] \cdot P\left[ {{\text{exceedance}}\left| {M = m\;} \right.} \right],$$

which is the *total expectation law*. In the equation, $P\left[ {{\text{exceedance}}\left| {M = m\;} \right.} \right] = 1/\left[ {1 + \exp \left( {- \alpha - \beta \cdot m} \right)} \right]$ is the probability that an earthquake of magnitude equal to *m* causes exceedance, which is evaluated in this section using logistic models. In fact, in the equation, $\alpha$ and $\beta$ are coefficients calibrated on data discussed above via logistic regressions. In particular, zero is attributed to the earthquakes not causing any exceedance and one to the earthquakes that cause exceedance (i.e., those discussed in the previous section).

Table 3 reports the coefficients of Eq. (2) for all the considered cases. It can be seen from the tables and from the figures that, for some cases, the regression is poorly constrained because of the paucity of data discussed in the previous section; this mostly applies, once again, for the lowest hazard class. Therefore, as a reference, logistic regressions pooling together the data from the different hazard classes are also given in the tables and in the figures (as solid black lines). The logistic regressions and data, as black bullets (note that each bullet can represent multiple earthquakes of the same magnitude), are shown in Fig. 3b,d for the two return periods considered.

**Table 3:** Logistic regression coefficients for probability to cause exceedance of the design actions as a function of magnitude.

	Tot	PGA ≤ 0.125 g	0.125 g < PGA ≤ 0.225 g	PGA > 0.225 g
**T**_**r**_** = 50 years**				
α	− 21.3762	− 14.4218	− 21.0635	− 28.4072
β	4.6960	3.2652	4.7754	6.0691
**T**_**r**_** = 475 years**				
α	− 21.9633	− 1.5694e+03	− 20.9460	− 25.1962
β	4.0836	356.3126	3.8658	4.6419

## Discussion

Models discussed above can be used to grossly predict the exceedance area of an earthquake of given magnitude occurring in Italy. To give an example, the fitting on the pooled data, not divided per hazard level, are considered. However, because the considered earthquakes are mostly from moderate-to-high hazard regions, the presented results can be considered mainly representative of this situation.

**Figure 4:**
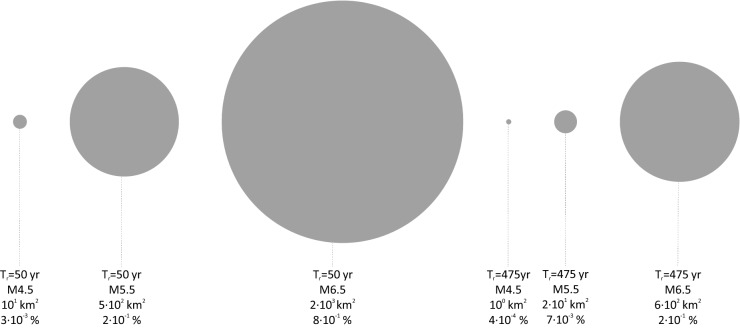
Exceedance area for structural design seismic action in terms of PGA with 50 and 475 years return period as per the models fitted in this study when magnitude is equal to 4.5, 5.5, and 6.5.

Figure 4 provides the PGA exceedance area, in squared kilometers and percentage of the Italian territory, obtained from Eq. (2) for three different magnitudes, 4.5, 5.5, and 6.5, for the two return periods considered in this study. These magnitude values are of interest for Italy because, earthquakes (i.e., mainshocks) with M ≥ 4.5, M ≥ 5.5 and M ≥ 6.5 have return period of a few months, a couple of years, and less than twenty years respectively, according to a recent seismic hazard model of Italy^14^. Moreover, 4.5 is about the minimum magnitude considered in the hazard assessment to determine the structural seismic actions in Italy.

The estimated exceedance area passes from a few square kilometers for the lowest magnitude to hundreds or thousands of kilometers, depending on the exceedance return period, for the largest magnitude and up to about one percent of the Italian territory. This is of engineering relevance as in these relatively vast areas around the source, structures could be systematically exposed to structural actions larger than those accounted for in design and should show enough seismic capacity to withstand them.

## Supplementary Information

Supplementary Information.

## Data Availability

ShakeMap data used in this study were obtained from http://shakemap.rm.ingv.it/shake4/, last accessed September 2021. Probabilistic seismic hazard analysis data at the basis of the Italian seismic code were obtained from http://esse1.mi.ingv.it/, last accessed September 2021. Exceedance areas computed to produce the results presented herein are available as supplemental information.
