# Supplementary material for: Empirical assessment of seismic design hazard’s exceedance area
Source: Sci Rep. 2021 Sep 22;11:18803. doi: 10.1038/s41598-021-98388-9 (PMC8458436; doi:10.1038/s41598-021-98388-9)
Supplement: Supplementary file 1 — Supplementary Information. [file 41598_2021_98388_MOESM1_ESM.pdf]

Empirical assessment of seismic design hazard’s exceedance area

by Iunio Iervolino, Antonio Vitale, and Pasquale Cito

Supplemental material: earthquakes causing exceedance for at least one return period from http://shakemap.rm.ingv.it/shake4/

										PGA exceedance Area [km^2]	
ID	Year	Month	Day	Time	Location	Lon [°]	Lat [°]	Depth [km]	M	T_r = 50 [yr]	T_r = 475 [yr]
1806269	2008	3	19	14:38:00	'4 km W Ischitella (FG)'	15.858	41.898	31.6	3.9	108.72	
1870169	2008	12	23	15:24:00	'5 km SE Neviano degli Arduini (PR)'	10.345	44.544	22.9	4.9	566.35	
1870749	2008	12	23	21:58:00	'5 km N Vetto (RE)'	10.355	44.527	23.7	4.3	141.94	
1895389	2009	4	6	01:32:00	'2 km SW L''Aquila (AQ)'	13.38	42.342	8.3	6.1	1201.39	80.88
1895879	2009	4	6	02:37:00	'6 km W L''Aquila (AQ)'	13.328	42.36	8.7	4.8	4.40	
1908319	2009	4	7	17:47:00	'1 km N Fossa (AQ)'	13.486	42.303	17.1	5.4	326.90	
1909629	2009	4	7	21:34:00	'3 km W L''Aquila (AQ)'	13.365	42.364	9.6	4.3	27.13	
1916789	2009	4	9	00:52:00	'5 km SE Capitignano (AQ)'	13.351	42.489	11	5.2	382.24	
1921649	2009	4	9	19:38:00	'4 km E Capitignano (AQ)'	13.35	42.504	9.3	5	196.83	
1940059	2009	4	13	21:14:00	'7 km E Capitignano (AQ)'	13.377	42.498	9	4.8	60.93	
2121679	2009	10	19	10:08:00	'4 km SE Gropparello (PC)'	9.772	44.814	23.6	3.9	47.87	
2257019	2010	9	17	12:20:00	'7 km E Foggia (FG)'	15.623	41.472	6	4.2	125.61	
2403649	2011	7	17	18:30:00	'1 km SW Ceneselli (RO)'	11.367	45.01	2.4	4.5	672.87	132.28
2406369	2011	7	25	12:31:00	'3 km S Giaveno (TO)'	7.365	45.016	11	4.3	376.14	
2445349	2011	10	29	04:13:00	'4 km SW Avio (TN)'	10.911	45.712	9.5	3.9	168.02	
772691	2012	5	20	02:03:00	'7 km NW Finale Emilia (MO)'	11.2635	44.8955	9.5	5.8	3253.21	1055.54
772991	2012	5	20	02:06:00	'7 km N San Felice sul Panaro (MO)'	11.165	44.9052	4.3	4.8	116.90	
773011	2012	5	20	02:07:00	'5 km NW Finale Emilia (MO)'	11.2703	44.8737	6.1	5	317.45	
773101	2012	5	20	02:11:00	'5 km NE Finale Emilia (MO)'	11.3412	44.8603	10.9	4.3	11.51	
774071	2012	5	20	03:02:00	'2 km NE San Felice sul Panaro (MO)'	11.152	44.8597	9.1	5	582.63	
783491	2012	5	20	13:18:00	'2 km SW Mirabello (FE)'	11.4407	44.8135	3.4	4.9	300.57	
783561	2012	5	20	13:21:00	'4 km E Finale Emilia (MO)'	11.3513	44.8332	8.3	4.1	123.64	
786001	2012	5	20	17:37:00	'4 km N Finale Emilia (MO)'	11.305	44.8653	5.4	4.2	176.87	
786031	2012	5	20	17:38:00	'6 km NW Finale Emilia (MO)'	11.2532	44.8798	3.7	4.6	155.07	
806721	2012	5	23	21:41:00	'4 km W Finale Emilia (MO)'	11.2438	44.8462	8.7	3.9	66.69	

816791	2012	5	25	13:14:00	'3 km NE Medolla (MO)'	11.0905	44.8688	4	3.7	41.84	
831851	2012	5	27	18:18:00	'4 km NE San Felice sul Panaro (MO)'	11.1642	44.8707	6	3.8	133.37	
841091	2012	5	29	07:00:00	'1 km SW Medolla (MO)'	11.0657	44.8417	8.1	5.6	2208.02	774.63
844021	2012	5	29	08:27:00	'2 km W Mirandola (MO)'	11.0415	44.8832	6	4.6	136.99	
844031	2012	5	29	08:25:00	'5 km SE Novi di Modena (MO)'	10.9475	44.8647	7.9	5	86.74	
844401	2012	5	29	08:40:00	'4 km SW San Possidonio (MO)'	10.9663	44.8695	4.1	4.1	66.13	
847781	2012	5	29	10:55:00	'3 km SW San Possidonio (MO)'	10.9795	44.8652	4.3	5.3	1017.15	124.95
847911	2012	5	29	11:00:00	'3 km SW San Possidonio (MO)'	10.9763	44.866	7.2	5.1	697.66	
847921	2012	5	29	11:00:00	'5 km SE Novi di Modena (MO)'	10.941	44.8558	8.7	5	328.24	
884491	2012	5	31	14:58:00	'3 km SE Rolo (RE)'	10.874	44.8663	8.2	3.7	104.36	
886121	2012	5	31	19:04:00	'2 km SW San Possidonio (MO)'	10.9843	44.8812	6.8	3.8	92.77	
908231	2012	6	3	19:20:00	'4 km W San Possidonio (MO)'	10.9502	44.886	8.7	4.7	746.56	146.77
955561	2012	6	12	01:48:00	'2 km E Novi di Modena (MO)'	10.9218	44.8908	8.3	3.9	187.36	
1360151	2012	10	3	09:20:00	'4 km E Sampeyre (CN)'	7.2343	44.5835	9.8	3.9	201.59	
1436611	2012	10	25	23:05:00	'3 km SE Mormanno (CS)'	16.0158	39.8747	9.7	5	307.26	
2479289	2012	1	24	23:54:00	'4 km E Negrar (VR)'	10.985	45.528	10.1	3.8	101.60	
2479469	2012	1	25	08:06:00	'4 km S Brescello (RE)'	10.51	44.871	29	4.9	709.80	124.81
1744261	2013	2	16	21:16:00	'4 km W Sora (FR)'	13.5697	41.7143	17.1	4.8	3.83	
1760951	2013	2	25	01:01:00	'4 km N Monastero di Lanzo (TO)'	7.4387	45.3415	15.7	3.3	56.36	
2151711	2013	6	21	10:33:00	'7 km NE Carrara (MS)'	10.1357	44.1308	7	5.1	467.38	
2181341	2013	6	23	15:01:00	'1 km W Minucciano (LU)'	10.2008	44.1683	9.2	4.4	52.72	
2243071	2013	6	30	14:40:00	'2 km SW Minucciano (LU)'	10.1867	44.1595	6.1	4.5	112.83	
2447831	2013	8	15	23:06:00	'2 km SE Gioiosa Marea (ME)'	14.9138	38.1627	24.8	4.2	34.19	
6984921	2016	7	30	20:21:00	'2 km SW Pinasca (TO)'	7.2122	44.9313	15.4	3.7	408.99	4.21
7073641	2016	8	24	01:36:00	'1 km W Accumoli (RI)'	13.2335	42.6983	8.1	6	2604.67	658.19
7076161	2016	8	24	02:33:00	'5 km E Norcia (PG)'	13.1507	42.7922	8	5.3	492.97	
7224451	2016	8	26	04:28:00	'3 km S Amatrice (RI)'	13.2915	42.6048	8.7	4.8	57.66	
7624821	2016	9	3	10:18:00	'6 km SE Castelsantangelo sul Nera (MC)'	13.2173	42.8607	8.3	4.3	9.36	
8663031	2016	10	26	17:10:00	'3 km SW Castelsantangelo sul Nera (MC)'	13.1243	42.8747	8.1	5.4	909.17	156.79
8663041	2016	10	26	17:10:00	'3 km SW Castelsantangelo sul Nera (MC)'	13.1272	42.8788	9.3	4.5	594.14	32.49
8669321	2016	10	26	19:18:00	'3 km S Visso (MC)'	13.0902	42.9048	9.6	5.8	2286.82	348.40
8669361	2016	10	26	19:18:00	'2 km W Castelsantangelo sul Nera (MC)'	13.1277	42.902	9.2	4.5	298.56	

8676191	2016	10	26	21:42:00	'4 km SW Castelsantangelo sul Nera (MC)'	13.1223	42.864	9.9	4.5	125.56	
8863681	2016	10	30	06:40:00	'4 km NE Norcia (PG)'	13.1092	42.8303	10	6.5	4494.19	1211.30
8865281	2016	10	30	07:13:00	'2 km W Accumoli (RI)'	13.2248	42.6943	10.5	4.2	29.45	
8882341	2016	10	30	12:07:00	'5 km SE Preci (PG)'	13.0757	42.8418	9.7	4.5	26.27	
8949131	2016	10	31	07:05:00	'6 km NE Norcia (PG)'	13.1263	42.8388	9.5	4	5.63	
9033461	2016	11	1	07:56:00	'5 km N Ussita (MC)'	13.1345	42.9902	8.3	4.8	85.38	
9166761	2016	11	3	00:35:00	'2 km S Pieve Torina (MC)'	13.0493	43.0277	8.1	4.7	285.32	
12695491	2017	1	18	09:25:00	'3 km NW Capitignano (AQ)'	13.2768	42.545	10	5.1	402.09	
12697591	2017	1	18	10:14:00	'2 km NW Capitignano (AQ)'	13.2838	42.531	9.6	5.5	723.84	0.63
12698071	2017	1	18	10:25:00	'3 km SW Capitignano (AQ)'	13.277	42.5033	9.4	5.4	363.85	
12707401	2017	1	18	13:33:00	'2 km N Barete (AQ)'	13.2747	42.4733	9.5	5	105.08	
18673351	2018	4	10	03:11:00	'2 km SW Muccia (MC)'	13.0365	43.0687	8.1	4.6	77.16	
20362671	2018	8	14	21:48:00	'2 km S Montecilfone (CB)'	14.8407	41.8877	19.2	4.6	141.18	
20375681	2018	8	16	18:19:00	'4 km SE Montecilfone (CB)'	14.8648	41.8742	19.6	5.1	409.50	
20845861	2018	10	6	00:34:00	'2 km S Ragalna (CT)'	14.9395	37.6088	4.5	4.6	8.11	
21266871	2018	12	24	16:50:00	'6 km W Zafferana Etnea (CT)'	15.039	37.716	2.2	4.3	6.07	
21285011	2018	12	26	02:19:00	'4 km NE Viagrande (CT)'	15.116	37.644	0.3	4.9	61.45	26.18
21355221	2019	1	8	23:50:00	'10 km NW Milo (CT)'	15.046	37.795	2.2	4.1	0.67	
22299431	2019	5	21	08:13:00	'3 km SE Barletta (BT)'	16.3003	41.2992	29.9	3.7	53.39	
23791601	2020	1	19	05:22:00	'2 km SW Neive (CN)'	8.1003	44.7168	11.6	3.1	10.29	
25871441	2020	12	29	14:36:00	'3 km W Salizzole (VR)'	11.0425	45.2442	9.4	3.9	161.47	
